# Is vitamin B12 deficiency a risk factor for gastroparesis in patients with type 2 diabetes?

**DOI:** 10.1186/s13098-023-01005-0

**Published:** 2023-03-01

**Authors:** Sally S. Ahmed, Hala A. Abd El-Hafez, Mohamed Mohsen, Azza A. El-Baiomy, Enas T. Elkhamisy, Mervat M. El-Eshmawy

**Affiliations:** 1 Internal Medicine Department, Faculty of Medicine, Mansoura Specialized Medical Hospital, Mansoura University, Box: 35516, Mansoura, Egypt; 2 Radiology Department, Faculty of Medicine, Mansoura University, Mansoura, Egypt; 3 Clinical Pathology Department, Faculty of Medicine, Mansoura University, Mansoura, Egypt

**Keywords:** Type 2 diabetes, Gastroparesis, Vitamin B12, Transabdominal ultrasonography

## Abstract

**Background:**

Diabetic gastroparesis is a severe diabetic complication refers to delayed gastric emptying in the absence of mechanical obstruction of the stomach. Vitamin B12 affects the dynamics of autonomic nervous system and its deficits has been linked to cardiovascular autonomic neuropathy therefore, vitamin B12 deficiency was hypothesized to be implicated in the development of diabetic gastroparesis. This study was conducted to explore the possible association between vitamin B12 deficiency and gastroparesis in patients with type 2 diabetes** (**T2D).

**Methods:**

A total of 100 T2D patients with diabetes duration > 10 years and 50 healthy controls matched for age and sex were recruited for this study. T2D patients were divided into 2 groups: patients with gastroparesis and patients without gastroparesis. The diagnosis of gastroparesis was based on Gastroparesis Cardinal Symptom Index (GCSI) Score ≥ 1.9 and ultrasonographic findings including gastric emptying ˂ 35.67% and motility index ˂ 5.1. Anthropometric measurements, plasma glucose, glycosylated hemoglobin (HbA1c), lipids profile, vitamin B12 and transabdominal ultrasonography were assessed.

**Results:**

The frequency of vitamin B12 deficiency in total patients with T2D was 35% (54.5% in patients with gastroparesis vs. 11.1% in patients without gastroparesis, P < 0. 001). Vitamin B12 level was negatively correlated with GCSI Score whereas, it was positively correlated with gastric emptying and motility index. Vitamin B12 deficiency was an independent predictor for gastroparesis in patients with T2D; it predicts gastroparesis at a cut off value of 189.5 pmol/L with 69.1% sensitivity and 64.4% specificity, P = 0.002.

**Conclusions:**

Beside the known risk factors of diabetic gastroparesis, vitamin B12 deficiency is an independent predictor of diabetic gastroparesis in patients with T2D.

## Background

Diabetic gastroparesis is a clinical syndrome characterized by delayed gastric emptying in the absence of mechanical obstruction of the stomach [1]. The characteristic symptoms of gastroparesis are early satiety, nausea, vomiting, bloating and upper abdominal pain [2] however, diabetic gastroparesis is often asymptomatic [3, 4]. Diabetic gastroparesis is not uncommon disease [5]; the reported prevalence from the tertiary referral centers is 10–30% of patients with type 2 diabetes (T2D) [6–8] whereas, the community prevalence using strict diagnostic criteria is 1.1% of patients with T2D [9]. Autonomic neuropathy, enteropathy and hyperglycemia are the most frequently implicated risk factors of diabetic gastroparesis [2, 10, 11]. Delayed gastric emptying leads to poor glycemic control and increased risk of hypoglycemia [12]. Indeed, gastroparesis significantly impairs quality of life [9] and is associated with morbidity and mortality [5, 13].

Recently, vitamin B12 levels have been found to be inversely related to glucose intolerance [14]. Additionally, vitamin B12 affects the dynamics of autonomic nervous system [15] and its deficits has been linked to cardiovascular autonomic neuropathy [16]. Vitamin B12 deficiency may be also implicated in the development of diabetic gastroparesis however, this association has not been yet investigated. Therefore, the aim of the present study was to explore the possible association between vitamin B12 deficiency and diabetic gastroparesis in patients with T2D.

## Methods

This study comprised 100 adult patients with T2D and 50 age- and sex-matched healthy controls. Patients with T2D were consecutively recruited from Diabetes Outpatient Clinic at Mansoura Specialized Medical Hospital, Mansoura University, Mansoura, Egypt. The inclusion criteria were patients with duration of T2D > 10 years and who had symptoms of gastroparesis [Gastroparesis Cardinal Symptom Index (GCSI) Score ≥ 1.9]. Patients with T2D were submitted for transabdominal ultrasonography accordingly, they divided into 2 groups: patients with gastroparesis (n = 55) and patients without gastroparesis (n = 45). Exclusion criteria were history of digestive tract surgery or prior gastric outlet obstruction, thyroid disease, liver & renal failure, neuropsychiatric disorder, connective tissue disorders, malignancies, pregnancy and participants taking vitamin B12 and alcohol. Drugs that could potentially interfere with gastrointestinal motility such as GLP-1 receptor agonists and the amylin analog, α glucosidase inhibitors, and opioid analgesic were also excluded. Healthy controls were recruited from the same geographic area with the same exclusion criteria.

All participants were subjected to a thorough medical history and underwent a clinical examination. Anthropometric measurements including height, body weight, body mass index (BMI) (kg/m^2^), and waist circumference (WC) were obtained using standardized techniques. The diagnosis of diabetic gastroparesis was based on the symptom validated questionnaire GCSI Score ≥ 1.9 and ultrasonographic findings including gastric emptying ˂ 35.67% and motility index ˂ 5.1. The cut-off point of gastric emptying and motility index were calculated from our study healthy controls as mean—2SD.

The GCSI Score consists of 9 symptoms covering 3 areas; nausea/vomiting subscale (3 symptoms: nausea, vomiting and retching), postprandial fullness/early satiety subscale (4 symptoms: stomach fullness, early satiety, postprandial fullness and loss of appetite) and bloating subscale (2 symptoms: bloating and stomach distension). All symptoms are rated from 0 to 5 over the prior 2 weeks [no symptoms = 0, very mild = 1, mild = 2, moderate = 3, severe = 4, and very severe = 5]. GCSI Score was calculated as the average of the 3 symptom subscales [17]. The clinical severity of gastroparesis was graded on a scale originally proposed by Abell et al. [18]; grade 1: mild gastroparesis (symptoms are relatively easily controlled and weight and nutrition can be maintained with a regular diet); grade 2: compensated gastroparesis (symptoms are partially controlled with the use of daily medications and nutrition can be maintained with dietary adjustments); grade 3: gastroparesis with gastric failure (uncountable refractory symptoms with frequent hospitalizations and/or inability to maintain nutrition via an oral route).

Vitamin B12 deficiency was defined as vitamin B12 levels below 125 pmol/L [16]. Peripheral neuropathy was diagnosed based on neuropathy disability and symptom scores [19, 20]. Diabetic nephropathy was diagnosed according to Umanath & Lewis [21]. Diabetic retinopathy was assessed through fundus examination.

### Laboratory assay

Fasting plasma glucose (FPG) and 2-h post prandial plasma glucose (PPG) were measured by commercially available kit, Cobas (Integra-400) supplied by Roche Diagnostics (Mannheim, Germany). Glycated hemoglobin (HbA1c) was estimated as an index of metabolic control on a DCA 2000 analyzer, fast ion exchange resin (Roche Diagnostic, Germany. Total cholesterol (TC), triglycerides (TGs) and high density lipoprotein cholesterol (HDL-C) were measured by commercially available kits (Cobas Integra-400). Low density lipoprotein cholesterol (LDL-C) was calculated according to Friedewald et al. [22]. Complete metabolic panel including ESR, complete blood count, renal, liver and thyroid function tests were also assessed. Serum vitamin B12 level was assayed by ELISA technique supplied by Bioassay technology.

### Ultrasonography assessment of gastric motility

**Fig. 1:**
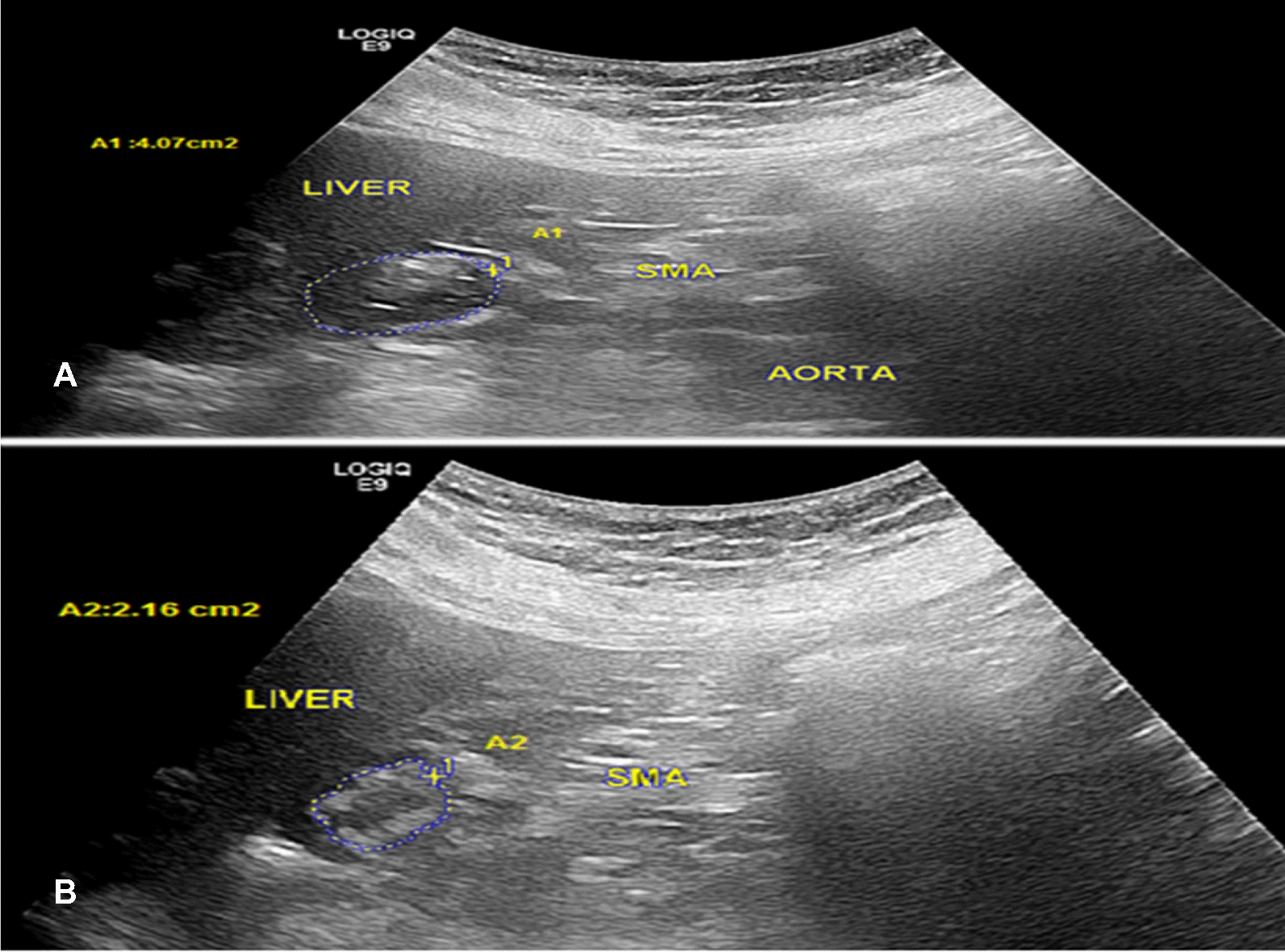
Vertically oriented ultrasonography demonstrates the relaxed phase at 1 min (**A**) and the contracted phase (**B**) at 3 min after ingestion of the meat soup. SMA: superior mesenteric artery, AO: aorta

After an overnight fasting, patients sat in a chair, leaned slightly backwards and drank 400 ml meat soup (54.8 kcal, 0.38 g protein and 0.25 g fat). An ultrasound probe was positioned vertically to permit simultaneous visualization of the gastric antrum, superior mesenteric artery and abdominal aorta for evaluation of the antral contractions Fig 1. The examination was conducted by GE LOGIQ E9 ultrasound machine with a 5 MHz convex probe [23]. The gastric emptying (%) was estimated as: ([antral area at 1 min] – [antral area at 15 min]/antral area at 1 min) × 100. The motility index was estimated by calculating the mean amplitude x frequency of contractions. The amplitude of antral contractions is the difference between the relaxed and contracted areas during a 3-min interval, divided by the relaxed area. The frequency of antral contractions is the number of contractions during a 3-min interval beginning 2 min after ingestion of soup. Ultrasonography was assessed by the same operator to minimize variation in the examination procedure. Fasting blood glucose was less than 275 mg/dl on the day of testing and patients had stopped prokinetic drugs 7 days before the procedure.

### Statistical analysis

This study was a pilot study with an initial sample size of 20 T2D patients who were excluded from the full scale study. The final calculated sample size was 97. Data entry and analysis were done by the SPSS statistical package (version 22, Armonk, NY: IBM Corp). The data were expressed as mean ± SD for continuous data, number and percent for categorical data and median (minimum–maximum) for skewed data. Student’s t and Mann–Whitney U tests were used to compare the 2 studied groups for parametric and non-parametric data, respectively. The chi-square and Fischer exact tests were performed to compare 2 or more groups of qualitative variables. The correlations of GCSI Score, gastric emptying and motility index with all other studied variables were analyzed by the Pearson and Spearman correlations analysis. Binary stepwise logistic regression analysis was used to predict the independent variables of binary outcome; the significant predictors in the univariate analysis were entered into the regression model. Receiver operating characteristic (ROC) curve was done to detect the level of vitamin B12 associated with gastroparesis in patients with T2D. Sensitivity, specificity, positive predictive value (PPV), negative predictive value (NPV), area under the curve (AUC) and 95% CI were evaluated. P ≤ 0.05 was considered as significant.

## Results

Patients with T2D had significantly higher BMI, WC, systolic & diastolic blood pressures (SBP & DBP), FPG, 2 h PPG, HbA1c, TC, TGs, LDL-C and lower HDL-C than did healthy controls. Among the studied participants with T2D, the median age of diabetes duration was 17 years, 63% had hypertension, 32% had retinopathy, 24% had diabetic nephropathy and 43% had neuropathy (18% moderate, 25% severe). With regard to anti-diabetic medications, 40% received insulin, 37% received oral anti-diabetic drugs and 23% received combined oral anti-diabetic drugs and insulin. Vitamin B12 was significantly lower in patients with T2D than in healthy controls, the frequency of vitamin B12 deficiency was 35% Table 1.

**Table 1:** Baseline characteristics of the study subjects

Characteristics	Patients with T2D (n = 100)	Healthy control (n = 50)	*P*-value
Age (years)	53.78 ± 9.65	53.36 ± 8.58	0.791
Gender			0.940
Women/Men	52/48	26/24	
Duration of diabetes (years)	17 (11–22)	–	–
Medications		–	–
Insulin alone	40 (40%)		
SU alone	17 (17%)		
SGLT2 inhibitors alone	7 (7%)		
TZD alone	6 (6%)		
Metformin alone	7 (7%)		
Insulin & metformin	11 (11%)		
Insulin & TZD	5 (5%)		
Insulin & SGLT2 inhibitors	7 (7%)		
BMI (Kg/m^2^)	36.47 ± 5.10	21.87 ± 2.01	< 0.0001*
WC (cm)	106.95 ± 21.21	79.32 ± 6.39	< 0.001*
SBP (mm Hg)	146.08 ± 13.81	109.52 ± 8.69	< 0.001*
DBP (mm Hg)	92.84 ± 8.89	71.92 ± 3.94	< 0.001*
History of hypertension n (%)	63 (63%)	–	–
Retinopathy n (%)	32 (32%)	–	–
Proliferative retinopathy n (%)	22 (22%)		
Non proliferative retinopathy n (%)	10 (10%)		
Nephropathy n(%)	24 (24%)	–	–
Peripheral neuropathy n (%)	43 (43%)	–	–
Moderate n (%)	25 (25%)		
Severe n (%)	18 (18%)		
FPG (mg/dl)	160.84 ± 33.26	84.44 ± 9.17	< 0.001*
2 h PPG (mg/dl)	256.71 ± 52.97	118.89 ± 11.85	< 0.001*
HbA1c (%)	9 ± 1.56	5.91 ± 0.30	< 0.001*
TC (mg/dl)	269.03 ± 49.50	166.46 ± 14.29	< 0.001*
TGs (mg/dl)	208.53 ± 46.17	92.76 ± 16.88	< 0.001*
LDL-C (mg/dl)	141.55 ± 32.79	77.78 ± 10.56	< 0.001*
HDL-C (mg/dl)	40.50 ± 5.40	59.28 ± 3.34	< 0.001*
Vitamin B12 level (pmol/L)	150 (47–569)	309 (127- 654)	< 0.001*
Vitamin B12 deficiency n (%)	35 (35%)	0	0.001*

*P is significant if ≤ 0.05

Data are expressed as means ± standard deviation, numbers, proportion or median (minimum–maximum), *T2D* type 2 diabetes**,** *BMI* body mass index, *WC* waist circumference, *SU* sulphonylurea, *TZD* thiazoldindiaone, *SGLT2* sodium glucose transporter, *SBP* systolic blood pressure, *DBP* diastolic blood pressure, *FPG* fasting plasma glucose, *2 h PPG* 2 h post prandial plasma glucose, *HbA1c* Hemoglobin A1c, *TC* total cholesterol, *TGs* triglycerides**,** *LDL-C* low density lipoprotein cholesterol, *HDL-C* high density lipoprotein cholesterol

T2D patients with gastroparesis had significantly longer diabetes duration, higher BMI, WC, SBP, DBP, FPG, HbA1c, TC, TGs and LDL-C compared with those without gastroparesis. The frequency of hypertension, proliferative retinopathy, nephropathy and peripheral neuropathy were significantly higher in patients with gastroparesis than in those without gastroparesis. Vitamin B12 level was significantly lower in patients with gastroparesis than in those without gastroparesis. The frequency of vitamin B12 deficiency was 54.5% in patients with gastroparesis and 11.1% in patients without gastroparesis, P = 0.001. The grade of gastroparesis in T2D patients was distributed as 78.2% for grade 1, 21.8% for grade 2 with no reported cases in grade 3. Gastric emptying and motility index were significantly lower in T2D patients with gastroparesis than in those without gastroparesis. With regard to GCSI Score, there was no significant difference between patients with and without gastroparesis, Table 2.

**Table 2:** Baseline characteristics of the studied T2D patients with and without gastroparesis

Characteristics	T2D patients with gastroparesis (n = 55)	T2D patients without gastroparesis (n = 45)	*P*-value
Age (years)	54.14 ± 9.27	53.33 ± 10.19	0.678
Gender			
Women/Men	29/26	23/22	0.872
Duration of diabetes (years)	17 (11–22)	11 (11–13)	0.001*
Medications			
Metformin	10 (18.2%)	8 (17.7%)	0.872
Non-metformin	45 (81.8%)	37 (82.3%)	0.639
BMI (Kg/m^2^)	38.75 ± 5.08	33.68 ± 3.56	0.001*
WC (cm)	117.59 ± 12.61	92.33 ± 3.46	0.001*
SBP (mm Hg)	150.93 ± 11.26	140.15 ± 14.42	0.001*
DBP (mmHg)	95.36 ± 9.18	89.75 ± 7.55	0.001*
History of HTN n (%)	41 (74.5%)	22 (48.9%)	0.008*
Retinopathy n (%)			
Proliferative	19 (34.5%)	3 (6.7%)	0.001*
Non proliferative	7 (12.7%)	3 (6.7%)	0.315
Nephropathy n (%)	20 (36.4%)	4 (8.9%)	0.001*
Peripheral neuropathy n (%)	35 (63.6%)	8 (17.8%)	0.001*
Moderate n (%)	20 (57.1%)	5 (62.5%)	0.004*
Severe n (%)	15 (42.9%)	3 (37.5%)	0.008*
FPG (mg/dl)	167.98 ± 32.35	152.11 ± 32.61	0.017*
2 h PPG (mg/dl)	263.90 ± 57.76	247.91 ± 45.56	0.134
HbA1c (%)	9.85 ± 1.66	7.95 ± 0.87	0.001*
TC (mg/dl)	308.40 ± 25.33	220.91 ± 20.50	0.001*
TGs (mg/dl)	218.89 ± 54.48	195.86 ± 29.25	0.012*
LDL-C (mg/dl)	155.07 ± 34.18	125.02 ± 21.86	0.001*
HDL-C (mg/dl)	40.01 ± 6.93	41.08 ± 3.88	0.327
Vitamin B12 level (pmol/L)	88 (47–567)	230 (55–569)	0.001*
Vitamin B12 deficiency n (%)	30 (54.5%)	5 (11.1%)	0.001*
Gastroparesis assessment parameters			
Grade of gastroparesis			
Grade 1	43 (78.2%)	–	–
Grade 2	12 (21.8%)		
Grade 3	0		
GCSI Score	2.6 (2–3)	2.3 (2–3)	0.703
Gastric emptying (%)	26.33 (19.6–29.9)	58.34 (46.48–78.12)	0.001*
Motility index	3.54 (2.12–4.12)	7.64 (6.35–9.56)	0.001*

*P is significant if ≤ 0.05

Data are expressed as means ± standard deviation, numbers, proportion or median (minimum–maximum), *T2D* type 2 diabetes, *BMI* body mass index, *WC* waist circumference, *SBP* systolic blood pressure, *DBP* diastolic blood pressure, *HTN* hypertension, *FPG* fasting plasma glucose, *2 h PPG* 2 h post prandial plasma glucose, *HbA1c* Hemoglobin A1c, *TC* total cholesterol, *TGs* triglycerides**,** *LDL-C* low density lipoprotein cholesterol, *HDL-C* high density lipoprotein cholesterol, *GCSI* Gastroparesis Cardinal Symptom Index

**Fig. 2:**
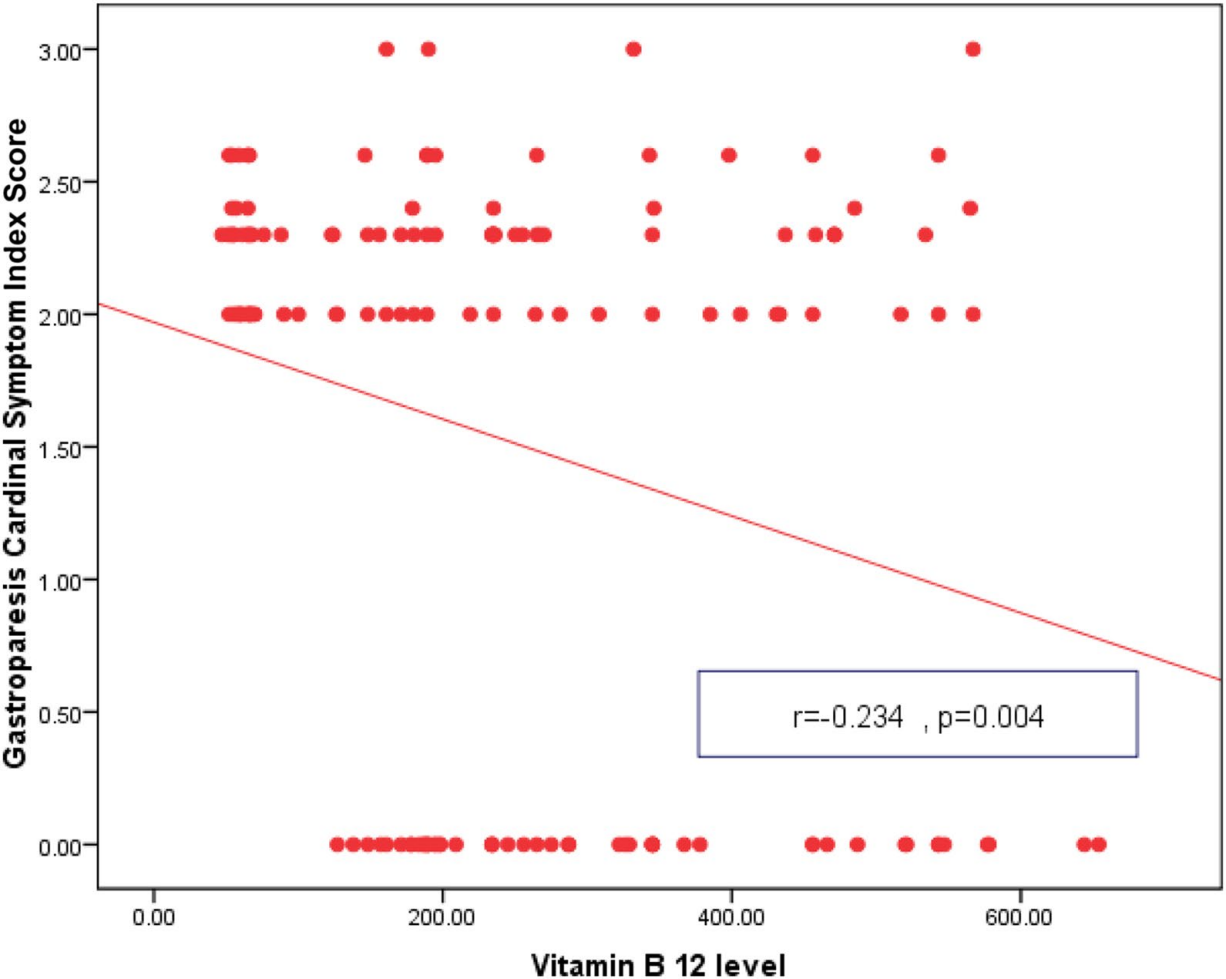
Correlation between vitamin B12 level and Gastroparesis Cardinal Symptom Index Score in T2D patients with gastroparesis

**Fig. 3:**
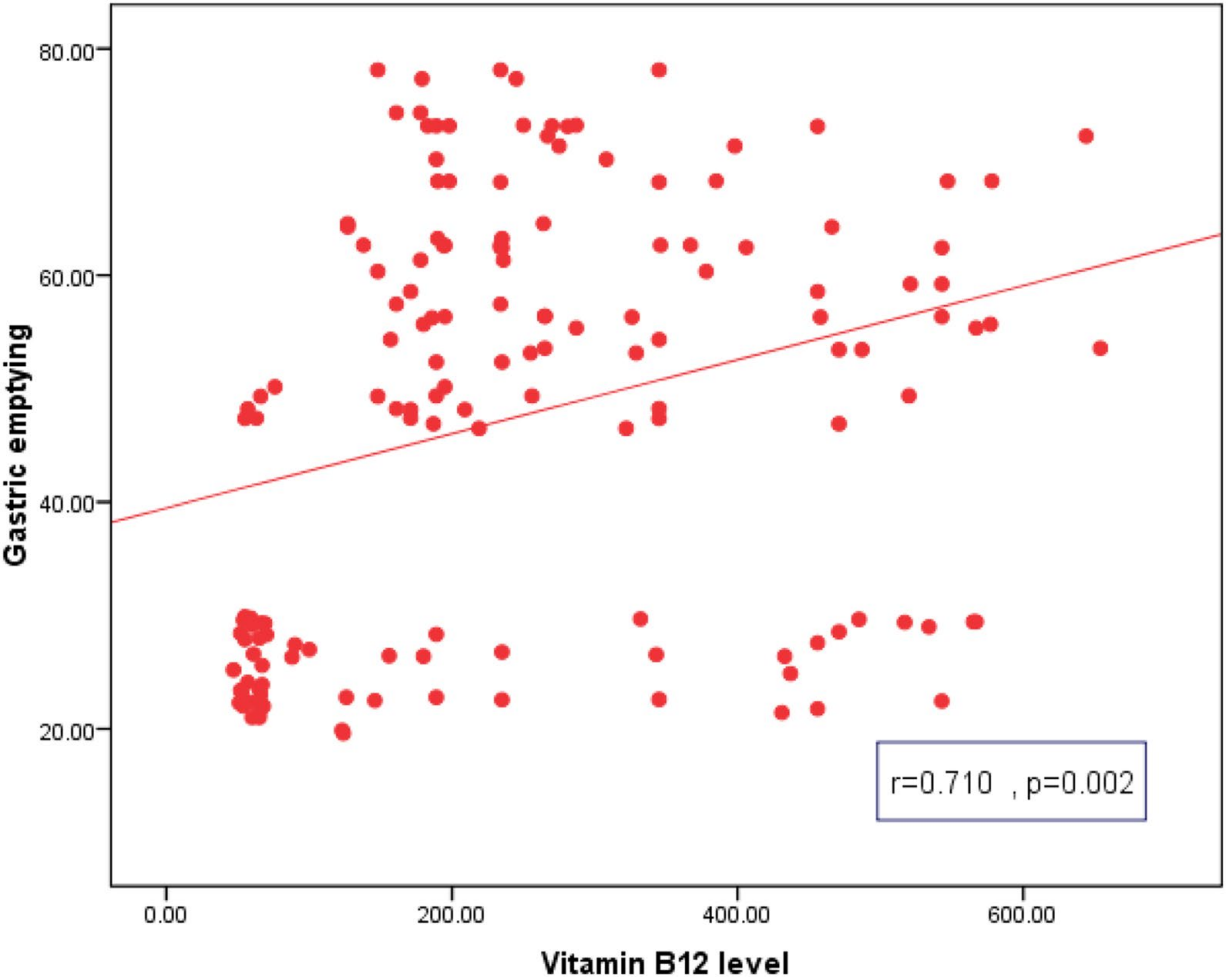
Correlation between vitamin B12 level and gastric emptying in T2D patients with gastroparesis

**Fig. 4:**
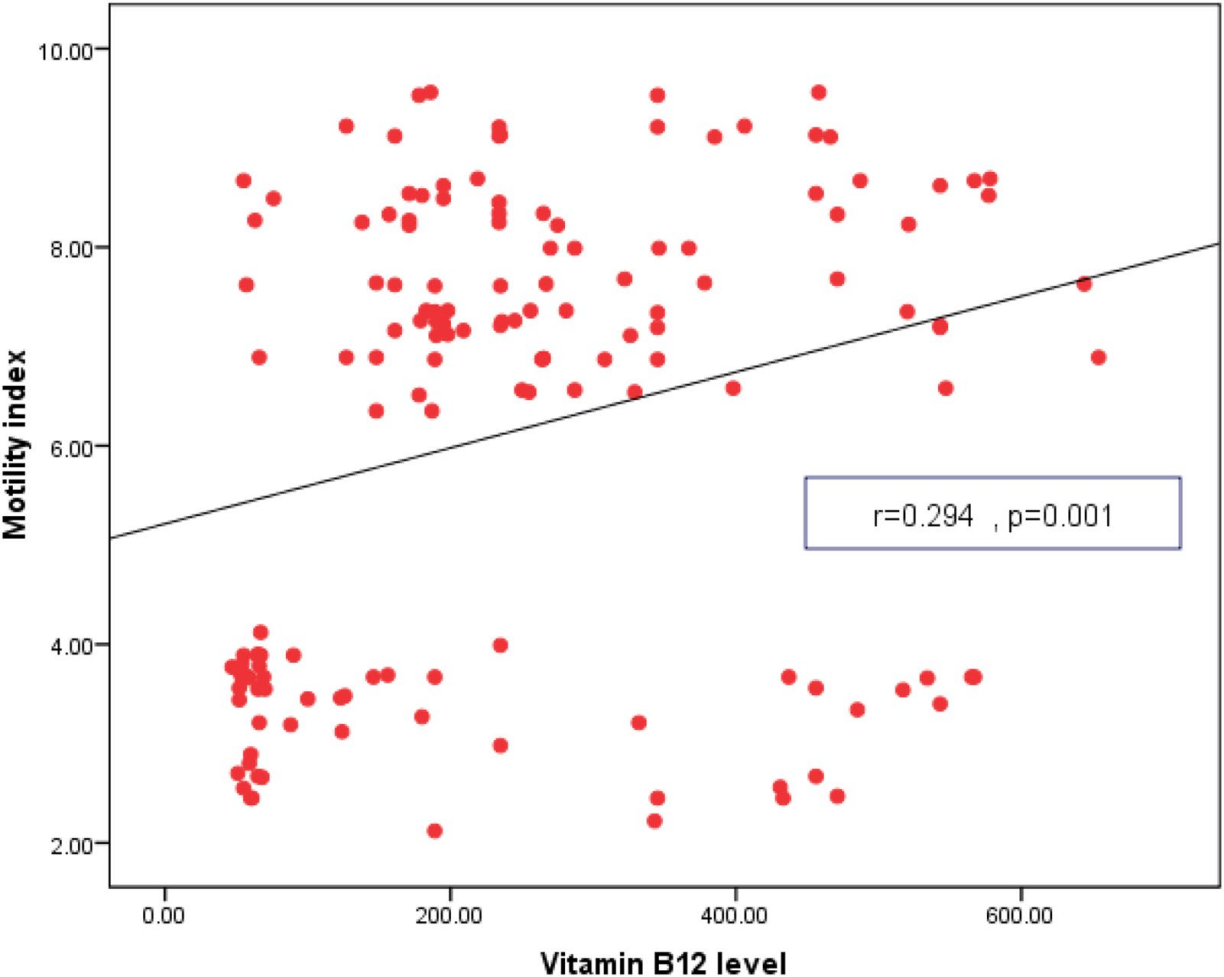
Correlation between vitamin B12 level and motility index in T2D patients with gastroparesis

GCSI Score was positively correlated with female sex, duration of T2D, BMI, SBP, DBP, retinopathy, nephropathy, neuropathy, FPG, HbA1c and grade of gastroparesis. Gastric emptying and motility index were negatively correlated with female sex, duration of T2D, BMI, SPB, DBP, retinopathy, nephropathy, neuropathy, FPG, HbA1c, TC, TGs and grade of gastroparesis. Table 3 Vitamin B12 levels were negatively correlated with GCSI Score and positively correlated with gastric emptying and motility index Figs 2, 3 and 4.

**Table 3:** Correlation of GCSI Score, gastric emptying and motility index, with other variables in T2D patients with gastroparesis

Variables	GCSI Score		Gastric emptying		Motility index	
	**r**	*P*-value	r	*P*-value	r	*P*-value
Age/years	0.135	0.18	− 0.125	0.21	− 0.245	0.58
Sex (female)	0.512	0.03*	− 0.614	0.02*	− 0.580	0.03*
T2D Duration	0.617	0.02*	− 0.715	0.01*	− 0.811	0.001*
BMI (kg/m^2^)	0.511	0.04*	− 0.545	0.03*	− 0.451	0.04*
WC (cm)	0.315	0.30	− 0.289	0.40	− 0.581	0.50
SBP (mm Hg)	0.680	0.02*	− 0.690	0.01*	− 0.582	0.02*
DBP(mm Hg)	0.598	0.04*	− 0.648	0.002*	− 0.521	0.02*
HTN	0.681	0.02*	− 0.692	0.01*	− 0.583	0.02*
Medications	0.135	0.18	− 0.125	0.21	− 0.245	0.58
Retinopathy	0.719	0.001*	− 0.695	0.002*	− 0.711	0.003*
Nephropathy	0.718	0.001*	− 0.689	0.02*	− 0.713	0.003*
Neuropathy	0.602	0.01*	− 0.631	0.003*	− 0.674	0.004*
FPG (mg/dl)	0.582	0.03*	− 0.697	0.02*	− 0.515	0.01*
2 h PPG(mg/dl)	0.112	0.60	− 0.251	0.40	− 0.241	0.10
HbA1c (%)	0.714	0.003*	− 0.805	0.01*	− 0.754	0.02*
TC (mg/dl)	0.036	0.79	− 0.513	0.04*	− 0.571	0.02*
TGs (mg/dl)	0.031	0.82	− 0.580	0.03*	− 0.697	0.02*
LDL-C (mg/dl)	0.258	0.50	− 0.198	0.60	− 0.215	0.70
HDL-C (mg/dl)	− 0.112	0.60	0.254	0.40	0.241	0.10
GCSI Score	–	–	− 0.540	0.69	− 0.028	0.84
Grade of gastroparesis	0.611	0.01*	− 0.628	0.01*	− 0.621	0.01*

*P is significant if ≤ 0.05

*T2D* type 2 diabetes, *GCSI* Gastroparesis Cardinal Symptom Index, *BMI* body mass index, *WC* waist circumference, *SBP* systolic blood pressure, *DBP* diastolic blood pressure, *HTN* hypertension, *FPG* fasting plasma glucose, *2 h PPG* 2 h post prandial plasma glucose, *HbA1c* Hemoglobin A1c, *TC* total cholesterol, *TGs* triglycerides**,** *LDL-C* low density lipoprotein cholesterol, *HDL-C* high density lipoprotein cholesterol

**Fig. 5:**
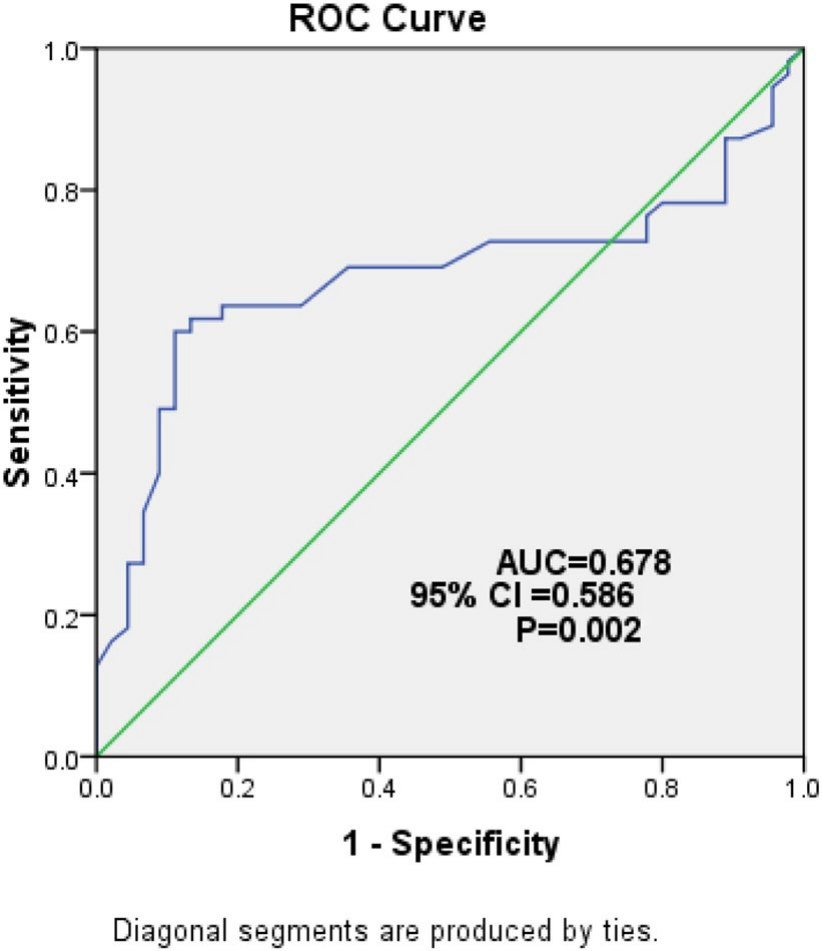
ROC curve

Female sex, duration of T2D, BMI, retinopathy, neuropathy, nephropathy, FPG, HbA1c, TC, TGs and vitamin B12 deficiency were significant positive predictors of gastroparesis in patients with T2D Table 4. The cutoff value of vitamin B12 level associated with gastroparesis was 189.5 pmol/I with 69.1% sensitivity, 64.4% specificity, 70.4% PPV and 63% NPV, AUC was 0.678, 95% CI (0.586–0.788), P = 0.002 Fig 5.

**Table 4:** Logistic regression analysis with gastroparesis as the dependent variable in patients with T2D

Variables	OR (95% CI)	β	*P*-value
Sex (Female)	1.25 (1.14–5.2)	0.251	0.02*
Duration of diabetes/years	16.95 (1.34–214.36)	0.251	0.02*
BMI (kg/m^2^)	2.80 (1.24–4.21)	1.250	0.03*
Retinopathy	3.10 (1.5–7.89)	0.124	0.01*
Nephropathy	2.95 (1.1–5.1)	0.114	0.02*
Peripheral neuropathy	2.80 (1.24–4.21)	0.254	0.03*
FPG (mg/dl)	1.06 (1.024–1.09)	1.250	0.01*
HbA1c %	17.07 (4.88–59.72)	3.254	< 0.001*
TC (mg/dl)	1.09 (1.03–1.16)	2.580	0.003*
TGs (mg/dl)	1.08 (1.02–1.14)	2.020	0.006*
Vitamin B12 deficiency	8.50 (3.5–15.89)	2.140	0.002*

*P is significant if ≤ 0.05

*OR* Odds Ratio, *CI* confidence interval**,** *T2D* type 2 diabetes, *BMI* body mass index, *FPG* fasting plasma glucose, *HbA1c* Hemoglobin A1c, *TC* total cholesterol, *TGs* triglycerides

## Discussion

In the current study, the diagnosis of diabetic gastroparesis was based on GCSI Score and transabdominal ultrasonography. Although gastric scintigraphy is the gold standard for diagnosis of gastroparesis [24, 25], ultrasonography is a radiation-free, readily available and a valid reliable imaging approach [26–28]. With transabdominal ultrasonography, gastric emptying and motility index were negatively correlated with female sex, duration of T2D, BMI, SPB, DBP, retinopathy, neuropathy, nephropathy, FPG, HbA1c, TC, TGs and grade of gastroparesis whereas, gastric emptying and motility index were not significantly correlated with GCSI Score. In line, Sogabe et al. [29] showed that gastric emptying and motility index values were significantly correlated with FPG. Authors concluded that the achievement of glycemic control improves both of gastric motility and gastrointestinal symptoms in patients with diabetic gastroparesis. Consistent with our results, Steinsvik et al. [30] found no significant associations between symptoms of gastroparesis and measurements of ultrasonography in patients with diabetic gastroparesis. In contrast, Darwiche et al. [31] found no significant associations between gastric emptying and the duration of diabetes, HbA1c, age or BMI; these incompatible findings are probably due to their small sample size.

In the present study, vitamin B12 level was significantly lower in patients with T2D than in healthy controls moreover, it was significantly lower in T2D patients with gastroparesis than in those without gastroparesis. Of interest, vitamin B12 was negatively correlated with GCSI Score whereas, it was positively correlated with gastric emptying and motility index. Additionally, vitamin B12 deficiency was an independent predictor for gastroparesis in patients with T2D. Vitamin B12 predicts gastroparesis at a cutoff value of 189.5 pmol/L with 69.1% sensitivity, 64.4% specificity, 70.4% PPV and 63% NPV, P = 0.002.

In the current study, the frequency of vitamin B12 deficiency in total patients with T2D was 35% (54.5% in patients with gastroparesis and 11.1% in patients without gastroparesis). However, our findings are much higher than estimates from a study conducted by Amjad et al. [32] where vitamin B12 deficiency was detected in 17.5% of patients with gastroparesis either diabetic or non-diabetic. The definition of vitamin B12 deficiency varies between studies; the cutoff point used in this study was 125 pmol/L which is comparable with Hansen et al. [16] however, it is low compared with what is used in other studies [33, 34]. The variability of cutoff limit for vitamin B12 could be explained by heterogeneity in age and race in the study populations.

Vitamin B12 deficiency is a major public health problem caused by age, consumption of vegetarian diets, malabsorption and drugs such as chronic use of omeprazole and metformin [35–38]. An adequate vitamin B12 is essential for the proper functioning of the nervous system through maintenance of the myelin nerve sheaths [39, 40] therefore, vitamin B12 deficiency induces neurological disorders such as peripheral neuropathy [41, 42]. Moreover, vitamin B12 is used in the treatment of peripheral neuropathy [43]. Furthermore, vitamin B12 affects the dynamics of autonomic nervous system [15] and a significant association between vitamin B12 deficiency and cardiovascular autonomic neuropathy has been recently reported [16]. In the light of these findings, we hypothesized that vitamin B12 deficiency may be also implicated in the development of diabetic gastroparesis. To our knowledge, this is the first study to indicate the independent association between vitamin B12 and diabetic gastroparesis.

In our study participants, the independent predictors of gastroparesis other than vitamin B12 deficiency were female sex, duration of diabetes, BMI, diabetic microvasascular complications, FPG, HbA1c, TGs and TC which are consistent with the existing literature [44–46].

In the current study, we did not observe an association between metformin treatment and diabetic gastroparesis. The increased frequency of vitamin B12 deficiency among patients with T2D taking metformin has been previously reported [47, 48], however this association depends on both the dose and the duration of treatment [49, 50]. It is believed that metformin induces vitamin B12 deficiency 5–10 years after treatment initiation due to late depletion of body storages [50]. In our study population, the duration of treatment was less than 5 years which may explain our finding.

Finally, in addition to sex, duration of diabetes, BMI, diabetic microvasascular complications, FPG, HbA1c, TGs and TC, vitamin B12 deficiency was an independent predictor of diabetic gastroparesis. From the previous discussion 2 raising questions arise which needs further studies. First, whether gastroparesis leads to vitamin B12 deficiency therefore, vitamin B12 may be a cause and a consequence of diabetic gastroparesis. Second, whether vitamin B12 supplementation can improve diabetic gastroparesis.

## In conclusion

Beside the known risk factors of diabetic gastroparesis, vitamin B12 deficiency is an independent predictor of diabetic gastroparesis in patients with T2D.

## Data Availability

All data generated or analyzed during this study are included in this published article.
